# Analysis of the Microbial Diversity and Characteristics of Fermented Blueberry Beverages from Different Regions

**DOI:** 10.3390/foods9111656

**Published:** 2020-11-12

**Authors:** Nan Hu, Ming Lei, Xiuli Zhao, Zhen Zhang, Ying Gu, Yan Zhang, Shuo Wang

**Affiliations:** 1 State Key Laboratory of Food Nutrition and Safety, Key Laboratory of Food Nutrition and Safety, Ministry of Education of China, Tianjin University of Science and Technology, Tianjin 300457, China; hunan1986@mail.tust.edu.cn (N.H.); leiming1993@mail.tust.edu.cn (M.L.); xiulizhao@mail.tust.edu.cn (X.Z.); zhangzhen1991@mail.tust.edu.cn (Z.Z.); guying@kust.edu.cn (Y.G.); 2 Tianjin Key Laboratory of Food Science and Health, School of Medicine, Nankai University, Tianjin 300071, China; yzhang@nankai.edu.cn

**Keywords:** blueberry, high-throughput sequencing, spontaneous fermentation, microbial diversity, antioxidant activity

## Abstract

In this study, high-throughput sequencing methods were used to analyze the composition and diversity of the microbial communities of three different traditional fermented blueberry beverages (Jiaosu A, Jiaosu B, and Jiaosu C) produced in three different regions. Lactic acid bacteria and yeast counts, total soluble solids, total titration acid, total phenols, total flavonoids, total anthocyanin, superoxide dismutase, and antioxidant activity were analyzed in all samples. The results showed that at the phylum level, the bacteria in all samples were predominantly Firmicutes and Proteobacteria, while the majority of fungus belonged to Ascomycota. At the genus level, *Lactobacillus*, *Gluconobacter*, and *Acetobacter* were the dominant bacteria, and *Dekkera* and *Issatchenkia* were the dominant fungi. Our data show that the lactic acid bacteria counts in Jiaosu A were the lowest of the three products, in the range of 4.31–10.9 log CFU/mL, while yeast counts ranged from 6.71 to 7.35 log CFU/mL. The antioxidant activities of Jiaosu C were greater than those of Jiaosu A and Jiaosu B, and Spearman correlation analysis showed that the relative abundance of *Lactobacillus* and *Dekkera* was significantly positively correlated with total phenolics, total anthocyanin, total flavonoids, and antioxidant index.

## 1. Introduction

Fermentation is one of the most commonly used ancient food preservation methods throughout human history and represents a valuable worldwide cultural heritage. In the absence of any refrigeration or other preservation methods, traditional fermentation can be used as a substitute method to produce staple foods for people [1]. Although a wide variety of foods have been developed along with the development of various new processing methods, food fermentation is still irreplaceable in food processing [2] and has evolved to produce foods with local characteristics, such as wine [3], Kimchi [4], bread [5], and Yucha (a kind of Chinese traditional fermented food) [6]. In 7000 BC, Chinese people started making fermented food using honey and fruits, a practice which remains common today [7]. Blueberry, a popular fruit rich in many bioactive molecules (especially polyphenols and flavonoids) [8], is a well-known antioxidant capable of scavenging free radicals, and this property is of great interest to the food industry [9]. At present, fermented blueberry beverages are favored for their rich nutritional content. In terms of taste, beverages based on fermented blueberry are also more readily accepted compared with those using fresh blueberry. Traditional fermented beverages are generally prepared by natural fermentation, a method that uses a combination of wild microbiota such as bacteria, yeasts, and molds [10,11]. It has been shown that fermentation of foods improves upon the nutritional value and health functions of the original unfermented raw materials [12]. Previous studies have shown that fermentation plays a critical role in enhancing antioxidation activities, suggesting a potential relationship between microorganism profiles and antioxidant properties [13,14,15,16,17,18,19].

At present, research on the microbial diversity of traditional fermented beverages is mainly based on the separation, purification, and identification of microbes, which rely on personal practical experience with many subjective factors [20]. Thus, traditional methods for the quantification of microbiota in foods generally do not accurately reflect microbial diversity due to the great variations in experimental conditions [21]. In recent years, high-throughput sequencing (HTS) has been widely used for studying microbial diversity in the context of food processing due to its advantages of higher throughput, greater accuracy, and requiring less time. Many researchers have used HTS technology to analyze bacterial and fungal diversity in traditional fermented foods [22,23].

In this study, high-throughput sequencing was used to compare and analyze the bacterial and fungal diversity of traditional fermented blueberry beverages from different regions of China, with the goal of identifying the dominant strains and analyzing their correlation with the antioxidant capacity of the fermented blueberry beverages and, further, investigating the fermentation mechanism underlying the traditional fermented products. This study provides relevant scientific data and a theoretical basis for the design of novel fermentation processes for producing blueberry beverages with high food quality and establishing safety controls.

## 2. Materials and Methods

### 2.1. Sample Collection

Traditional fermented blueberry beverages Jiaosu A, Jiaosu B, and Jiaosu C were respectively purchased from the Yichun, Heihe, and Jiagedaqi regions of Heilongjiang Province of China in August 2017.

### 2.2. DNA Extraction, 16S rRNA, and Internal Transcribed Spacer (ITS) Sequencing

The traditional fermented blueberry beverage samples were sent to Novogene bioinformatics institute (Beijing, China) and the sample DNA of each was extracted using the cetyltrimethylammonium bromide (CTAB) method [24]. The primer pairs 515F/806R and ITS 5-1737F/ITS 2-2043R were used for PCR amplification of the bacterial 16S V4 and fungal ITS 1 regions, respectively, and the resulting PCR products were used to construct the high-throughput sequencing library, followed by single-ended sequencing based on the Ion S5 XL platform (Thermo Fisher Scientific, Waltham, MA, USA).

### 2.3. Enumeration of Lactic Acid Bacteria and Yeast

The procedure for enumeration of lactic acid bacteria [6] and yeast [25] is as follows: A 10-fold gradient dilution was prepared by adding a 25 mL sample to 225 mL sterile saline. The sample solutions with appropriate dilution were chosen and inoculated in MRS agar plates and Rose Bengal agar plates to obtain viable counts of lactic acid bacteria (LAB) and yeast, respectively; the inoculum size was 1 mL. The MRS agar plates were incubated at 37 °C for 48 h under anaerobic conditions, and the Rose Bengal agar plates were incubated at 28 °C for 5 days. Ternary samples of different regions were collected and measured.

### 2.4. Determination of pH Value, Total Soluble Solids and Titratable Acidity

The pH value was measured using a pH meter (FE 28 pH meter from Mettler-Toledo, Shanghai, China). The total soluble solids (TSS) content was determined using a sugar meter. The total titratable acidity (TTA) was determined according to the IFU 3 method of the International Federation of Juice Producers (Paris, France). The sample was titrated with 0.1 mol/L NaOH until pH 8.1 and results are expressed as mg anhydrous citric acid (ACA) per mL [26].

### 2.5. Determination of Total Phenolic Content

The total phenolic content (TPC) in the fermented blueberry beverage samples was determined using the Folin–Ciocalteu method [27]. The specific methods were as follows: 1 mL of appropriately diluted fermented blueberry beverage sample was added to 5 mL 0.2 N Folin–Ciocalteu’s reagent and homogenized with 4 mL of 7.5% (*w*/*v*) sodium carbonate aqueous solution, then incubated at 40 °C for 5 min in the dark. The blank sample was H_2_O. The absorbance was measured at 765 nm using a spectrophotometer (Thermo fisher scientific, Waltham, Massachusetts, USA). The results are expressed as mg gallic acid equivalents (GAE) per 100 mL of sample. Each extract sample was analyzed in triplicate.

### 2.6. Determination of Total Flavonoid Content

For estimation of the total flavonoid content (TFC) [28], the specific methods were as follows: 1 mL of diluted sample was homogenized with 0.4 mL of 5% NaNO_2_ and allowed to react at room temperature for 6 min. Then, 0.4 mL of 10% AlCl_3_ was added followed by incubation at room temperature for 6 min. After that, 3 mL of 1 M NaOH was added and incubated at room temperature for 5 min. The mixed reaction solution was drawn into a 10 mL volumetric flask and diluted with ddH_2_O to the scale line. After 15 min of incubation, the absorbance of the pink mixture was measured at 510 nm versus the prepared blank (ddH_2_O). The results are expressed as mg rutin equivalents (RE) per 100 mL of sample. Each extract sample was analyzed in triplicate.

### 2.7. Determination of Total Anthocyanin Content

The total anthocyanin content (TAC) was estimated using a pH differential method [29]. Volumes of 0.5 mL of diluted sample were added to buffer solutions of pH 1.0 and 4.5 at constant volumes of 10 mL and allowed to react in the dark for 30 min. The absorbance was measured at 510 and 700 nm. The TAC is expressed as mg cyanidin-3-glucoside equivalents (C3GE) per 100 mL of sample. Each extract sample was analyzed in triplicate.

### 2.8. Determination of Antioxidant Capacity

The ferric reducing antioxidant power (FRAP) was measured using the Total Antioxidant Capacity (T-AOC) test kit (Solarbio, Beijing, China). The reagents were prepared according to the instruction manual. A 6 μL aliquot of diluted sample (distilled water in the case of the blank) was added to 180 μL of the mixture solution and 18 μL of ddH_2_O, followed by mixing for 10 min. The absorbance value was measured at a wavelength of 593 nm.

The effect of fermented blueberry beverage on 2,2-diphenyl-1-picrylhydrazyl (DPPH) radicals was estimated according to the method of Gow-Chin Yen et al. [30]. Diluted samples (40–200 μL) were added to 3 mL of 0.1 mM DPPH solution. The mixture was left to stand at room temperature for 30 min; the absorbance of the resulting solution was measured at 517 nm versus the prepared blank (ddH_2_O). Vitamin C solution was used as a control. Each extract sample was analyzed in triplicate.

The 2,2’-azinobis-(3-ethylbenzthiazoline-6-sulphonate) (ABTS) radical anions were used according to the method of Oszmianski et al. [31]. ABTS stock solution (7 mM concentration) and 2.45 mM potassium persulfate were mixed at a ratio of 1:1 (*v*/*v*) and left out of the light at room temperature for 16 h. The mixture was diluted with phosphate buffer to an absorbance of 0.7 (± 0.02) at 734 nm to produce ABTS radical cations (ABTS^+^). A 0.1 mL aliquot of sample diluent was added to 3.9 mL ABTS radical cation solution and left to react at 37 °C for 6 min. The absorbance was measured at 734 nm versus the prepared blank (ddH_2_O). Vitamin C solution was used as a control. Each extract sample was analyzed in triplicate.

### 2.9. Determination of Superoxide Dismutase

The superoxide dismutase (SOD) activity was determined using the total SOD activity detection kit according to the WST-8 method (Beyotime, Shanghai, China) [32]. Twenty microliters of appropriately diluted samples were added to 160 μL WST-8/enzyme working solution and 20 μL reaction starting solution and incubated at 37 °C for 30 min; we then determined the absorbance value at 450 nm. Blank 1 was 20 μL SOD test buffer added to 160 μL WST-8/enzyme working solution and 20 μL reaction starting solution; Blank 2 was 40 μL SOD test buffer added to 160 μL WST-8/enzyme working solution; and Blank 3 was 20 μL of appropriately diluted sample added to 20 μL SOD test buffer and 160 μL WST-8/enzyme working solution.

### 2.10. Data Analysis

Cutadapt [33] was used first to remove low-quality reads, and the data for each sample were then divided from the obtained reads based on their barcodes. Raw reads were obtained by trimming off barcode and primer sequence for quality control. Read sequences that removed chimeric sequences were compared and detected using the species annotation database and the UCHIME algorithm [34]; the chimeric sequences were finally removed [35] to give the final clean reads. Uparse software [36] was used to cluster all clean reads of all samples, and sequence clustering was converted into operational taxonomic units (OTUs) by default with 97% identity. Qiime software (Version 1.9.1, Rob Knight, University of California, San Diego, USA) was used to calculate the observed OTUs, Chao1, Shannon, and Simpson indexes. The rank abundance curves and principal coordinate analysis (PCoA) graphs were plotted using R software (Version 2.15.3, Ross Ihaka & Robert Gentleman, University of Auckland, Auckland, New Zealand).

All experiments were performed at least in triplicate; the results are reported as the mean values ± standard deviation. Analysis of variance (ANOVA) by Duncan’s test (*p* < 0.05) was conducted using SPSS 20.0 software (SPSS Inc., Chicago, IL, USA).

## 3. Results

### 3.1. Abundance and Diversity of Bacteria and Fungi in Traditional Fermented Blueberry Beverages

**Figure 1:**
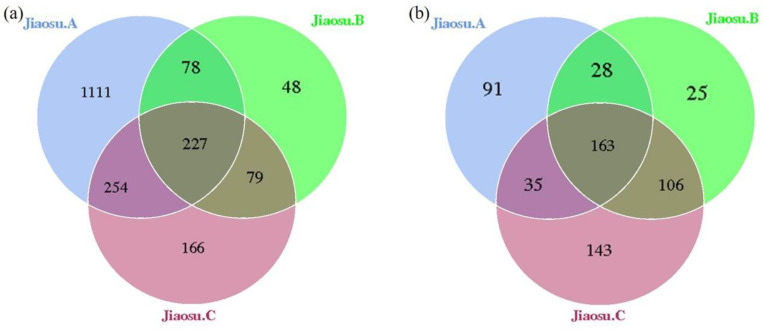
Venn plot of bacterial (**a**) and fungal (**b**) operational taxonomic units (OTUs) in all samples.

After all the samples were clustered based on 16S rDNA and ITS amplification sequence information, the proportions of OTU clustering data that could be constructed in the original data of bacteria and fungi were 91.60% (669,249/730,655) and 84.34% (527,103/624,950), respectively (Table 1). The total clean reads of all samples were clustered, revealing 1964 and 591 OTUs of bacteria and fungi, respectively; of these, 11.56% (227/1964) and 27.58% (163/591) were common to all three samples (Figure 1), indicating that there were significant differences among the three groups of samples. The number of bacterial OTUs in Jiaosu A (1670) was much higher than that in Jiaosu B and Jiaosu C (Figure 1a), while the identified fungi OTUs were quite similar for the three groups (Figure 1b). Further species annotation on the OTU representative sequences showed that the number of bacteria species in Jiaosu A was much higher than that in the other two groups, while the number of fungal species obtained from the three groups of samples was quite similar (Table 1). The Chao 1 index and number of species for bacteria in Jiaosu A were much higher than those for fungi, indicating that Jiaosu A had more rare species of bacteria, and that the species abundance of bacteria in Jiaosu A was greater than that of fungi. The Simpson and Shannon indexes reflect the distribution uniformity of microorganisms, and the larger the value, the better the distribution uniformity of microorganisms. The Simpson index values of bacteria in Jiaosu A and Jiaosu C were similar, while that of Jiaosu B was slightly lower. With regard to fungi, the Simpson index value for Jiaosu C was the lowest, and were similar for the other two groups. Jiaosu A had the highest bacterial Shannon index value, and Jiaosu B had the highest fungal Simpson index value (Table 1).

### 3.2. Bacterial and Fungal Community Distribution in Traditional Fermented Blueberry Beverage Samples

**Figure 2:**
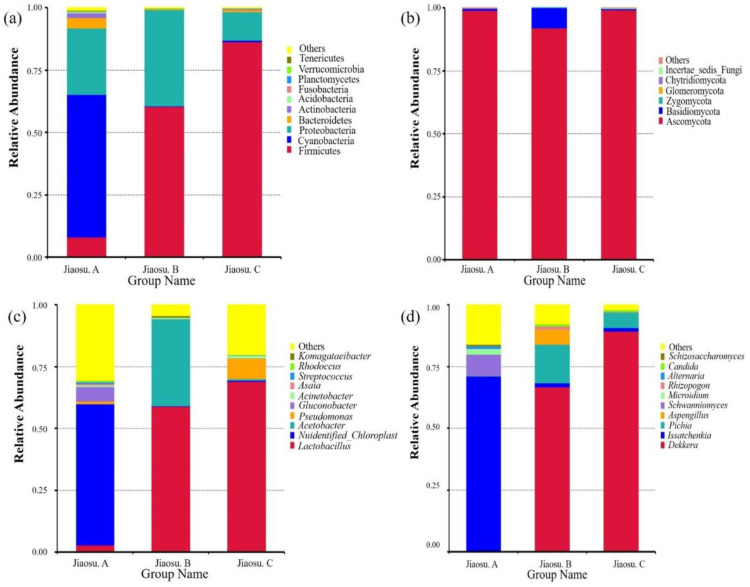
Relative abundance of bacteria (**a**) and fungi (**b**) at the phylum level and bacteria (**c**) and fungi (**d**) at the genus level in all samples. Note: The figure shows only the top 10 for each category (phyla or genera) in terms of relative microorganism abundance.

Annotation information at the phylum and genus levels along with the relative abundance for the top ten is shown in Figure 2. The horizontal annotation values of bacteria and fungi in all samples were 44 and 7, and the horizontal annotation values of genera were 524 and 82, respectively. The phyla represented by bacteria of relatively high abundance were Firmicutes, Cyanobacteria, Proteobacteria, Bacteroidetes, and Actinobacteria (abundance of ≥1% in at least one sample) (Figure 2a). The relative abundance of Cyanobacteria in Jiaosu A ranked first, accounting for 57.29%. Firmicutes species had the highest relative abundance in Jiaosu B and Jiaosu C, accounting for 60.45% and 86.39%, respectively. In addition, the relative abundance of Proteobacteria in the three groups was also relatively high, ranging from 10% to 40%. The genera with higher abundance of bacteria were *Lactobacillus*, *Unidentified_Chloroplast*, *Acetobacter*, *Pseudomonas*, and *Gluconobacter* (abundance of ≥1% in at least one sample) (Figure 2c). Among them, 57.22% of OTUs in Jiaosu A were annotated as *unidentified_Chloroplast*. Moreover, *Gluconobacter* and *Lactobacillus* had slightly higher abundance levels of 5.95% and 2.73%, and the rest were all less than 1%. In Jiaosu B, the abundance of *Lactobacillus* reached 58.88%, but the relative abundance of *Acetobacter* was also relatively high, accounting for 35.37%. In Jiaosu C, *Lactobacillus*, with a relative abundance of 69.14%, was the dominant genus, while *Pseudomonas* represented a low relative abundance of 8.52%.

At the phylum level, in terms of relative abundance among fungi, more than 90% of the fungal community was Ascomycota. In addition, the relative abundance of Basidiomycota in Jiaosu B was 7.95%, and the values for the remaining phyla were less than 1% (Figure 2b). The genera with higher abundance among fungi were *Dekkera*, *Issatchenkia*, *Pichia*, *Aspergillus*, *Schwanniomyces*, and *Microidium* (abundance of ≥1% in at least one sample) (Figure 2d). In Jiaosu A, *Issatchenkia* ranked first with a relative abundance of 70.54%, followed by *Schwanniomyces* with a relative abundance of 8.64%. In Jiaosu B, the relative abundance of *Dekkera* was 66.66%, followed by *Issatchenkia* and *Pichia* at 17.61% and 15.57%, respectively. Meanwhile, *Dekkera* also ranked the highest in Jiaosu C with relative abundance of 89.33%, followed by *Pichia* with relative abundance of 6.26%. In terms of fungal diversity across all samples, the dominant genera were *Dekkera* and *Issatchenkia*, in that order.

### 3.3. Comparison of Bacterial and Fungal Communities in Traditional Fermented Blueberry Beverage Samples

**Figure 3:**
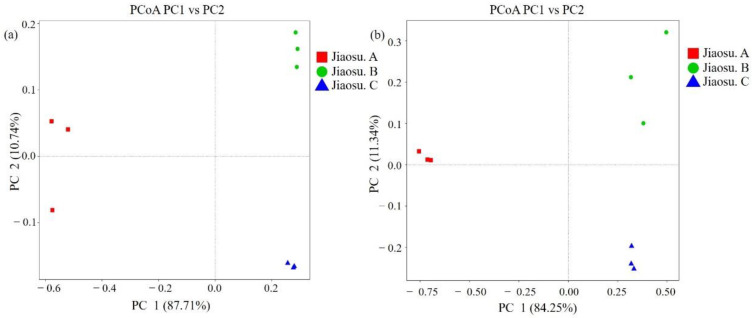
Principal coordinate analysis (PCoA) of the bacterial (**a**) and fungal (**b**) communities in all samples. Note: PC1 and PC2 were used to plot all PCoA results.

The weighted UniFrac distance and unweighted UniFrac distance were used to conduct principal coordinate analysis to determine the similarity of the constituent structures of microbial species in all samples (Figure 3). As shown in Figure 3, there were significant differences in the composition and structure of microbial species among the three groups of samples. The similarity of the bacterial composition of Jiaosu C was the best, while that of Jiaosu A was the worst, containing an outlier sample (Figure 3a). The compositions of fungal species in Jiaosu A and Jiaosu C were similar, while the distance among the three samples in Jiaosu B was much greater, indicating that the fungal species were less similar (Figure 3b).

**Figure 4:**
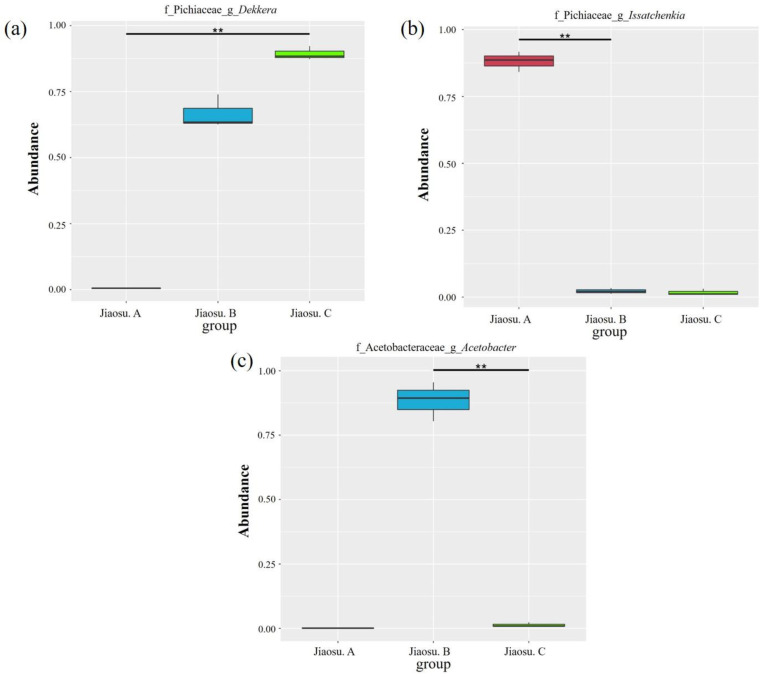
Diagrams of the distribution boxes of fungi (**a**,**b**) and bacteria (**c**) at the genus level. Note: “f”, family; “g”, genus; “**” indicates significant differences between groups (*p* < 0.01).

To examine variations in the microorganisms present in all the samples from different regions, the MetaStat method was used to test the relative abundance data between different groups with *p* values. The distribution box diagram of the top 10 at the genus level is shown in Figure 4. Significant differences in microorganisms among the top 10 at the genus level were found for *Dekkera*, *Acetobacter* and *Issatchenkia*.

### 3.4. LAB, Yeast Count, and Antioxidant Indicators

The LAB count for Jiaosu A was only 4.31 log CFU/mL, while the counts for the other two groups were relatively close, ranging between 9.86 and 10.9 log CFU/mL. In terms of the yeast count, ranging from 6.71 to 7.35 log CFU/mL, the number for Jiaosu A was slightly higher than that of the two groups. The TSS value ranged from 2.4 to 3.5 °Bx and was higher in Jiaosu A than in the other two groups. The pH value and TTA showed an opposite pattern. All the other indicators showed the same pattern, Jiaosu C > Jiaosu B > Jiaosu A, with significant differences (*p* < 0.05), with the exception of DPPH and SOD (Table 2).

**Figure 5:**
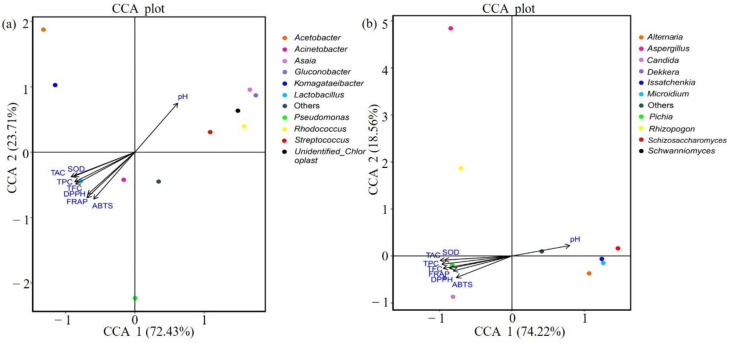
Redundancy analysis with environmental factors of bacteria (**a**) and fungi (**b**) in all samples. Note: The figure shows only the top 10 genera in terms of relative microorganism abundance.

**Figure 6:**
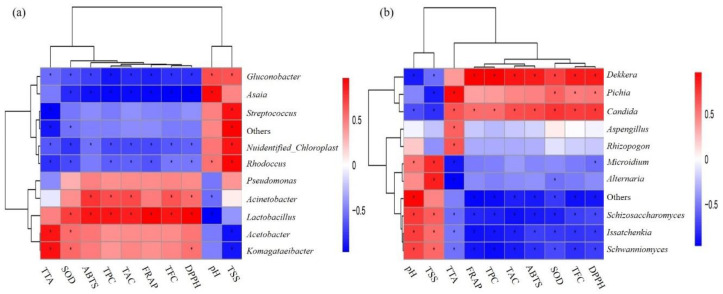
Spearman analysis with environmental factors of bacteria (**a**) and fungi (**b**) in all samples. Note: The figure shows only the top 10 genera in terms of relative microorganism abundance.

Canonical correlation analysis (CCA) was used to analyze the relationship between microorganisms and antioxidant indexes (Figure 5). When the ray angle between indicators is an acute angle, it means that the two indicators have a positive correlation; an obtuse angle indicates a negative correlation. Antioxidant indexes were thus positively correlated with anthocyanin, total phenolic, and total flavone contents and negatively correlated with pH value. In terms of the relationships between bacteria and physical and chemical indexes, *Lactobacillus* were negatively correlated with pH value but positively correlated with antioxidant-related indexes and anthocyanin content (Figure 5a). *Dekkera* and *Pichia* also showed similar correlations with pH value and antioxidant indexes (Figure 5b). Further, Spearman correlation coefficient analysis showed that among the top 10 species in terms of relative abundance of fungi and bacteria, *Lactobacillus*, *Dekkera*, and *Pichia* showed significant positive correlations with antioxidant indexes (Figure 6).

## 4. Discussion

The diverse distribution of bacteria and fungi in fermented foods has great significance in determining product quality. The diversity of microorganisms in traditional fermented foods is a decisive factor controlling the quality and nutritional value of subsequent products. In this study, 16S rRNA and ITS sequencing were used to comprehensively characterize the diversity of microbial communities in three traditional fermented blueberry beverages from different regions. First of all, at the level of bacterial genus, the main fermentation bacteria in the Jiaosu B and Jiaosu C sample group were *Lactobacillus*, which is the fermentation strain commonly used in the fermentation of fruits and vegetables [37,38]. A small amount of *Pseudomonas*, which was reported to be the main microorganism in mustard fermentation products [39], was detected in the Jiaosu C sample group. *Acetobacter* also had a high relative abundance in the Jiaosu B sample group. Traditional fermentation is mainly anaerobic fermentation; however, *Acetobacter* was found to occur in high abundance in the Jiaosu B sample even though it is an aerobic microorganism. The highest relative abundance in the Jiaosu A sample was attributed to *Unidentified_Chloroplast*, indicating that the number of bacteria in the Jiaosu A sample was low, which was also confirmed by the bacterial live cell count results (Table 2). In addition, in the Jiaosu A sample group, only *Lactobacillus* and *Gluconobacter* had a relative abundance of more than 1%. Although the number of bacteria in the Jiaosu A sample group was low, the dominant bacteria species could be clearly detected and identified. The reason why the bacteria in Jiaosu A did not proliferate rapidly enough to reach a numerical advantage may be due to some uncontrollable factors in the natural fermentation process. The existence of *Gluconobacter* and *Acetobacter* in the Jiaosu A and Jiaosu B samples may be mainly caused by the ethanol produced in the process of fungal fermentation and the amount of carbon source added in the early stage of fermentation, as reflected in the results for fungal count (Table 2).

Based on the information for fungi at the genus level, the highest relative abundance in Jiaosu A was attributed to *Issatchenkia*, which can produce higher levels of ethanol in fermented sweet sorghum stem [40]; its occurrence has also been reported in the early stage of fruit wine fermentation for the production of ethanol, and it can reduce the malic acid content [41,42]. Using a mixture of *Issatchenkia* and *Saccharomyces* with bacteria to ferment arbutus wine can effectively shorten the main fermentation time and reduce the production of capric acid while resulting in the production of a variety of esters [43]. The highest relative abundance of fungi in the Jiaosu B and Jiaosu C samples was attributed to *Dekkera*, which was one of the earliest yeasts to be isolated from fermented foods and beverages [44]. It is commonly used with *Saccharomyces cerevisiae* in wine fermentation and has been identified as a unique microbe present during fermentation of the characteristic type of red wine in some regions [45]. *Pichia* also had good relative abundance in all samples. *Pichia* is widely used in drinks containing ethanol and could produce some flavor esters with *S. cerevisiae* during fermentation [46]. The relatively high abundance of fungi in these traditional fermented blueberry beverages from three different regions has been confirmed in various traditional fermented foods and beverages [47,48].

On the whole, although bacteria and fungi are symbiotic in the whole fermentation system, bacteria still enjoy a certain advantage in terms of cell number (Table 2). These results may be due to the fact that the autolysis of yeast releases a large number of nutrients that supplement the fermentation with carbon and nitrogen sources, thus promoting the growth of other microorganisms [49]. At the same time, the existence of yeast contributes to the formation of product flavor and aroma [50]. The abundant growth of bacteria can specifically inhibit the production of fungal toxins to some extent, which can effectively improve food safety [51].

LAB and yeast have a certain positive effect on the antioxidant capacity of products [15,19], which was also confirmed in this study (Figure 6). It is well documented that the traditional blueberry beverages possess various antioxidants, including phenolics [52], flavonoids [53], anthocyanins [54], and superoxide dismutase [55]. Fermentation can lead to the breakdown of the cell wall and release of a variety of bioactive compounds [56], including the molecules mentioned above. It has been reported that yeast and LAB co-fermented milk can produce antioxidant peptides [57]. The contents of soluble phenols, flavonoids, quercetin, and kaempferol in the product were remarkably improved in guava leaf tea when subject to mixed fermentation with *Monascus anka* and *Saccharomyces cerevisiae* [58]. In addition, many LAB have enzymatic and non-enzymatic antioxidative mechanisms [59]. Therefore, the antioxidant capacity of traditional fermented blueberry beverages may also be derived from the action of the microorganisms involved in fermentation or their metabolites [13,16], which can exert antioxidant activities. For example, exopolysaccharides produced by *Lactobacillus plantarum* exhibit high antioxidant activity [60].

## 5. Conclusions

In this study, we analyzed the characteristics and microbial diversity in traditional fermented blueberry beverages from three major production regions in Heilongjiang, China. Our results clearly showed that the traditional fermented blueberry beverages from three different production areas showed distinct microbial populations, though LAB and yeast, including *Lactobacillus*, *Dekkera*, and *Issatchenkia*, were the most abundant microorganisms in the traditional fermented blueberry beverages and participated in the whole fermentation process. In addition, the abundance of *Lactobacillus* and *Dekkera* was potentially related to the antioxidant activity of the traditional fermented blueberry beverages. Given that the quality of traditional fermentation products is greatly affected by variety in the raw materials, production processes, and environmental conditions (such as temperature, humidity, and season), understanding the composition of the major microbial populations involved in the process of traditional blueberry fermentation, as revealed in this study, will guide the design of novel processes for the production of improved fermented blueberry beverages.

## Figures and Tables

**Table 1:** Sequence abundance and microbial diversity of all samples.

Sample ID	Raw Reads		Clean Tags		Number of OTUs		Observed Species		Chao1 Index		Simpson Index		Shannon Index	
	Bacteria	Fungi	Bacteria	Fungi	Bacteria	Fungi	Bacteria	Fungi	Bacteria	Fungi	Bacteria	Fungi	Bacteria	Fungi
Jiaosu A	238,455	221,394	213,279	183,055	1670	317	888	209	1005.125	232.498	0.868	0.928	4.907	4.737
Jiaosu B	251,629	199,545	235,353	168,265	432	322	287	248	329.996	277.954	0.775	0.914	3.477	5.054
Jiaosu C	240,571	204,011	220,616	175,779	726	447	408	286	438.773	323.8	0.883	0.668	4.024	3.554

Note. OTUs, operational taxonomic units.

**Table 2:** Total lactic acid bacteria (LAB) and yeast enumeration and antioxidant characteristics of all samples.

Samples	Jiaosu A	Jiaosu B	Jiaosu C
**LAB enumeration (log CFU/mL)**	4.31 ± 0.23 ^a^	9.86 ± 0.10 ^b^	10.90 ± 0.05 ^c^
**Yeast enumeration (log CFU/mL)**	7.35 ± 0.22 ^c^	6.71 ± 0.13 ^a^	6.95 ± 0.08 ^b^
**TTA (ACA, mg/mL)**	6.61 ± 0.10 ^a^	7.36 ± 0.10 ^c^	7.00 ± 0.11 ^b^
**TPC (GAE, mg/mL)**	3.23 ± 0.15 ^a^	5.05 ± 0.25 ^b^	5.85 ± 0.21 ^c^
**TFC (RE, mg/mL)**	0.44 ± 0.01 ^a^	0.60 ± 0.03 ^b^	0.68 ± 0.02 ^c^
**TAC (C3GE, mg/mL)**	0.32 ± 0.003 ^a^	0.55 ± 0.002 ^b^	0.60 ± 0.004 ^c^
**ABTS (IC50, mg/mL)**	2.40 ± 0.02 ^a^	2.45 ± 0.02 ^b^	2.54 ± 0.02 ^c^
**DPPH (IC50, mg/mL)**	1.73 ± 0.08 ^a^	2.50 ± 0.39 ^b^	2.53 ± 0.17 ^b^
**FRAP (U/mL)**	13.14 ± 0.28 ^a^	15.85 ± 0.55 ^b^	19.55 ± 0.16 ^c^
**SOD (U/mL)**	162.72 ± 22.90 ^a^	263.55 ± 19.61 ^b^	285.27 ± 4.91 ^b^
**TSS (°Bx)**	3.5 ± 0.23 ^b^	2.4 ± 0.06 ^a^	2.6 ± 0.06 ^a^
**pH**	3.92 ± 0.03 ^c^	3.55 ± 0.04 ^a^	3.77 ± 0.05 ^b^

Note. Values represent mean ± standard deviation (*n* = 3). Different superscript letters indicate significant differences between groups (*p* < 0.05). TSS, total soluble solids; FRAP, ferric reducing antioxidant power; SOD, superoxide dismutase; TTA, total titratable acidity, the results expressed as mg anhydrous citric acid (ACA) per mL; TPC, total phenolic content, the results expressed as mg gallic acid equivalents (GAE) per mL; TFC, total flavonoid content, the results expressed as mg rutin equivalents (RE) per mL; TAC, total anthocyanin content, the results expressed as mg cyanidin-3-glucoside equivalent (C3GE) per mL; ABTS and DPPH, the results expressed as mg Vitamin C (VC) per mL, IC50, concentration required to obtain 50% of the maximum antioxidant effect.
